# Next generation clinical guidance for primary care in South Africa – credible, consistent and pragmatic

**DOI:** 10.1371/journal.pone.0195025

**Published:** 2018-03-30

**Authors:** Shingai Machingaidze, Karen Grimmer, Quinette Louw, Tamara Kredo, Taryn Young, Jimmy Volmink

**Affiliations:** 1 Cochrane South Africa, South African Medical Research Council, Cape Town, South Africa; 2 European and Developing Countries Clinical Trial Partnership (EDCTP), Cape Town, South Africa; 3 Physiotherapy Department, Faculty of Medicine and Health Sciences, Stellenbosch University, Cape Town, South Africa; 4 Centre for Evidence-Based Health Care (CEBHC), Stellenbosch University, Cape Town, South Africa; The Chinese University of Hong Kong, HONG KONG

## Abstract

**Background:**

Agreed international development standards underpin high quality *de novo* clinical practice guidelines (CPGs). There is however, no international consensus on how high quality CPGs should ‘look’; or on whether high quality CPGs from one country can be viably implemented elsewhere. Writing d*e novo* CPGs is generally resource-intensive and expensive, making this challenging in resource-poor environments. This paper proposes an alternative, efficient method of producing high quality CPGs in such circumstances, using existing CPGs layered by local knowledge, contexts and products.

**Methods:**

We undertook a mixed methods case study in South African (SA) primary healthcare (PHC), building on findings from four independent studies. These comprised an overview of international CPG activities; a rapid literature review on international CPG development practices; critical appraisal of 16 purposively-sampled SA PHC CPGs; and additional interrogation of these CPGs regarding how, why and for whom, they had been produced, and how they ‘looked’.

**Results:**

Despite a common aim to improve SA PHC healthcare practices, the included CPGs had different, unclear and inconsistent production processes, terminology and evidence presentation styles. None aligned with international quality standards. However many included innovative succinct guidance for end-users (which we classified as evidence-based summary recommendations, patient management tools or protocols). We developed a three-tiered model, a checklist and a glossary of common terms, for more efficient future production of better quality, contextually-relevant, locally-implementable SA PHC CPGs. Tier 1 involves transparent synthesis of existing high quality CPG recommendations; Tier 2 reflects local expertise to layer Tier 1 evidence with local contexts; and Tier 3 comprises tailored locally-relevant end-user guidance.

**Conclusion:**

Our model could be relevant for any resource-poor environment. It should reduce effort and costs in finding and synthesising available research evidence, whilst efficiently focusing scant resources on contextually-relevant evidence-based guidance, and implementation.

## Background

The theory and practice of clinical practice guideline (CPG) writing has evolved over the past 35 years. This reflects growing sophistication in clinical epidemiology, technical writing and application of new technologies to evidence synthesis. Changing CPG definitions over this time period highlight this evolution. The Institute of Medicine (IOM) described CPGs in 1990 as ‘*systematically developed statements to assist practitioner and patient decisions about appropriate health care for specific clinical circumstances*’http://ebn.bmj.com/content/2/2/38.full—ref-1 (p. 38) [1]. This definition was updated in 2011 to emphasise the importance of rigorous methodology, suggesting that ‘*Clinical guidelines are statements that include recommendations intended to optimize patient care that are informed by a systematic review of evidence and an assessment of the benefits and harms of alternative care options*’ (p. 15) [2]. The current CPG definition focuses on implementation, stating that ‘*Guidelines are a convenient way of packaging evidence and presenting recommendations to healthcare decision makers*‘ (p. 6) [3].

Over the past 35 years, internationally-respected CPG development groups have been established, such as (but not limited to) National Institute for Health and Care Excellence (NICE) [4] and the Scottish Intercollegiate Guidelines Network (SIGN) [5]. These groups regularly produce CPGs for a wide range of topics and health conditions. There are also internationally-respected internet-based repositories of CPGs, such as the USA Agency for Healthcare Research and Quality (AHRQ) Guidelines Clearing House [6], Guidelines International Network (G-I-N) [7], and the World Health Organisation (WHO) [8]. Many professional associations also host CPGs on their websites or libraries. Consequently, it is not difficult to find answers for most healthcare questions, from at least one international CPG repository.

However, the explosion of CPG activity internationally has led to lack of standardisation about how CPGs are written and the evidence presented. Comparing two or more CPGs on the same topic, even from respected international CPG developers, immediately highlights differences [4–8]. Whilst this diversity is an enviable product of independent, international intellectual endeavours in the area, it also makes it increasingly difficult for end-users to decide on which CPGs to use. End-users can variably include healthcare providers, policy-makers, health managers and planners, insurers and/ or patients. Rethinking CPG writing and presentation to increase end-user uptake was a focus of the 2016 Guidelines International Network (G-I-N) conference in Philadelphia, United States of America. There were many presentations about making CPGs more relevant to end-users, and in believably presenting CPG recommendations in ways that encourage evidence uptake [9–11]. While there is now international consensus on key quality components of CPGs [12–14], there is no international consensus on how CPGs should ‘look’, as the term ‘CPG’ can refer to many different ways of presenting evidence summaries.

In this paper, we describe steps we took to develop a theoretical model aimed at efficiently improving the quality and uptake of contextually-relevant CPGs. Our research grew from a commitment to improve CPG quality, relevance and uptake in environments with limited resources for *de novo* (new) CPG writing. This research focused on South African (SA) primary healthcare (PHC). Resources for SA CPG writing and evidence implementation have been constrained for three decades, despite the escalating and currently-unmet need for best-evidenced, standardised guidance to redress the increasing prevalence of communicable and non-communicable diseases [15–17]. It is thus essential that scant health resources are put to best use to efficiently implement best available, locally-relevant evidence into SA PHC practices.

PHC became a SA government priority in the 1994 National Health Plan [18, 19]. PHC providers (GPs, nurses, allied health practitioners) care for South Africans over their lifespan, with PHC being the usual point of entry into the SA healthcare system [15, 17]. In SA, clinical guidance for PHC conditions has been developed independently by different groups, including the National and Provincial Departments of Health at district and facility levels, and by professional societies [20]. There is no central, nationally-recognized and/ or accepted CPG development unit. Given the escalating numbers of South Africans requiring PHC for increasingly complex, comorbid health conditions [15–19], it is imperative that SA PHC providers are conversant with, and able to access and apply, current best evidence to diagnose and care for the patients presenting at PHC facilities [21].

## Methods

### Ethics approval

Ethics approval was provided by the South African Medical Research Council (HREC EC002-2/3014).

### Purpose

To produce a simple theoretical model to underpin the efficient production of contextually-relevant good quality CPGs for resource-constrained environments.

### Model development strategy

To identify and address gaps between international CPG standards, and the way evidence was presented in SA PHC CPGs, whilst recognising and addressing local contexts, implementation barriers and end-user needs [15–19].

### Research design

**Fig 1:**
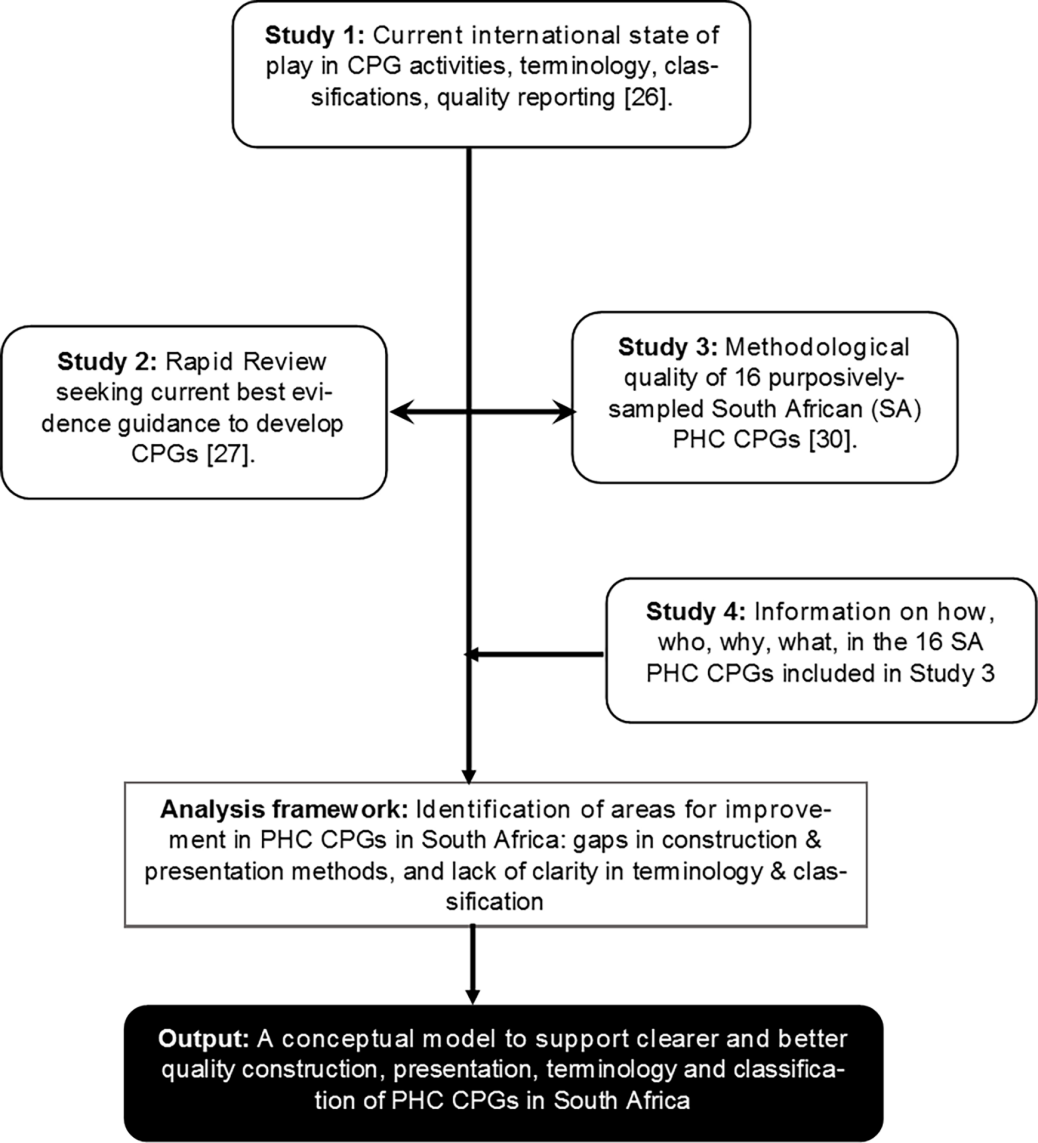
Case study approach.

We conducted a mixed methods case study [22, 23] which amalgamated findings from four independent studies conducted as part of a large research project into SA PHC CPGs (Project SAGE (South African Guidelines Excellence)) [24]. The Good Reporting of A Mixed Methods Study (GRAMMS) reporting framework guided the research [25]. S1 Appendix outlines how our research met the GRAMMS reporting criteria. Fig 1 describes the case study approach.

In Study 1 (conducted October 2014- January 2015), we deconstructed the ‘mysteries’ of international CPG activities and nomenclature to establish a comprehensive current ‘state of play’ [26]. Study 1 set the scene for Project SAGE by describing the complexities of writing and implementing good quality, locally-relevant CPGs [24]. Study 1 comprised a content review of documents (and their references lists), found on publically-available international CPG websites [4–8]. We reviewed reports, publications, blogs, podcasts and interviews, conference presentations and position statements. Using content analysis, we identified themes, gaps in activity, quality measures and current theory and practice in CPG writing.

In Study 2 (conducted December 2014- March 2015), we undertook a systematic rapid review of English language literature reporting on methods of CPG development [27]. The search was conducted from January 1^st^, 1990 to December 31^st^, 2014, to include publications since the IOM CPG definition was proposed [1]. Databases covering health and social sciences literature and grey literature were searched, comprising Biomed Central Gateway; CINAHL database; Cochrane Library; EMBASE; ERIC; Health Source (Nursing / Academic Edition); PsychInfo; Scopus; Web of Knowledge; World Health Organisation (WHO); Scottish Intercollegiate Guidelines Network (SIGN); National Guideline Clearinghouse (NGC); UK Department of Health publications; UK National Institute for Health and Clinical Excellence Guidelines (NICE); and Google Scholar. Search terms were customised to each database, and included synonyms of ‘CPGs’, ‘development’ and ‘methodological quality’. The Medline search strategy and ‘hits’ are provided in S2 Appendix as an example. In line with rapid review practices, we sought current systematic reviews as the best available evidence, and assessed their quality with the Critical Appraisal Skills Program (CASP) systematic review tool [28, 29].

In Study 3 (February 2015- April 2015), we used the AGREE II instrument (Appraisal of Guidelines for Research and Evaluation) to assess the methodological quality of 16 purposively-sampled SA PHC CPGs [30]. AGREE II is an internationally-accepted critical appraisal tool for researchers and developers, which assesses CPG quality in domains of CPG scope and purpose, stakeholder involvement, rigour of development, clarity of presentation, applicability of the CPG to its intended setting, and editorial independence [13]. The selected CPGs included documents produced in SA, outlining diagnosis, treatment, and/or clinical management of commonly-presenting conditions in SA PHC settings [15, 17, 19]. These comprised asthma, chronic obstructive pulmonary disease (COPD), hypertension, Type 2 diabetes, human immune-deficiency syndrome (HIV) (children, adults, prevention of mother-to-child transmission (PMTCT)), tuberculosis (TB), malaria, maternal care, Primary Care (PC 101) and integrated management of childhood illnesses (IMCI). We also included the Essential Drug List (EDL) which is used by SA healthcare providers to guide medicines choice.

For Study 4, we extracted and collated further details from the Study 3 CPGs [30], regarding who developed and published them, how the evidence was reported, who the end-users were, the CPG scope, purpose and aims, and the ways in which guidance was presented.

### Data analysis

For Studies 3 and 4, the AGREE II methodological quality scores were calculated as:

- median and interquartile ranges (IQR) for the six quality domains across the 16 CPGs; and
- overall quality scores for each CPG. This calculation was undertaken by adjusting the AGREE II domain scoring rubric to include all 23 AGREE II items. Whilst this is not the usual AGREE II scoring approach [13], it provided an overall estimate of methodological quality for comparison across the CPGs.

The findings from Studies 3 and 4 were compared with international CPG terminology and production methods (Studies 1 and 2), to identify gaps, possible reasons for them, and solutions to improve SA PHC CPG quality.

## Results

### Study 1

Our summary of the current ‘state of play’ of international CPG activity highlighted not only variability in CPG writing processes and terminology, but also fragmentation in how information was reported [26]. This highlighted the emphasis internationally on CPG development, and identified research gaps in evidence updating, as well as ways of transferring evidence developed in one setting to different settings. We identified the difficulty that inexperienced CPG writers and end-users might have, in understanding and applying current CPG writing processes and terminology in their own contexts. This relates to confusion surrounding the terminology and construction methods for CPGs; variable ways in which evidence is reported and recommendations framed; how to deal with inconsistent evidence and its interpretation; and presentation of end-user guidance.

### Study 2

The rapid literature review identified 1501 potentially-relevant records (1355 after removal of duplicates). After screening for relevance, 83 potentially-relevant records were considered in full text. These included one recent comprehensive systematic literature review (102 references) [14], and 82 primary literature and grey literature sources (comprising observational and descriptive studies, and guideline development manuals). The systematic review [14] scored 92% for methodological quality [29, 31], and was thus deemed to be the best available evidence [28]. It included 80 of the 82 identified primary evidence sources. The two not-included articles were published in the 1990s, and their exclusion was unlikely to have influenced the systematic review findings. The systematic literature review proposed 18 domains of quality construction, with the domains 8, 10, 11, 12, 13 and 14 focusing on the methodology of a good quality CPG [14]. These are bolded in the list below. The quality domains comprise 1. organisation, budget, planning and training; 2. priority setting; 3. guideline group membership; 4. establishing guideline group processes; 5. identifying target audience and topic selection; 6. consumer and stakeholder involvement; 7. conflict of interest considerations; ***8*. question generation;** 9. considering importance of outcomes and interventions, values, preferences and utilities; **10. deciding what evidence to include and searching for evidence; 11. summarising evidence and considering additional information; 12. judging quality, strength of certainty of a body of evidence; 13. developing recommendations and determining their strength; 14. wording of recommendations and considerations about implementation, feasibility and equity;** 15. reporting and peer-review; 16. dissemination and implementation; 17. evaluation and use; 18. updating [14].

### Study 3

Whilst the SA PHC guidance documents were labelled ‘CPGs’, they had variable and generally low scores in the AGREE II methodological quality domains [30], and none looked similar to CPGs produced by international developers [4,5]. Considering the median (IQR) AGREE II domain scores across the 16 included CPGs, the domains with highest scores were clarity of presentation (69% (44–94%)); and scope and purpose (55% (19–92%)). The remaining domains scored poorly (stakeholder involvement (22% (0–64%)); applicability (13% (0–83%)); rigour of development (4% (0–30)); and editorial independence (0% (0–29%)). These findings differed little from an earlier review of the methodological quality of CPGs produced in selected African countries for five priority diseases [31], suggesting that SA CPGs generally compared poorly with international CPG quality construction indicators. Despite this, many of the SA PHC CPGs contained innovative and contextually-relevant guidance. This potentially reflected their common purpose of providing simple guidance for healthcare providers to improve uptake of evidence into local PHC practices [15–17]. Thus rigour of development and comprehensive reporting of CPG methodology may have been of less concern to the SA PHC CPG writers, than providing easy-to-follow guidance about how care should be provided to patients in PHC settings [27, 30].

### Study 4

There was no standard approach to how the evidence underpinning the SA PHC CPG recommendations had been identified, collated, evaluated or presented, and there was no evidence of utility for end-users in PHC settings. Table 1 reports details on the CPG developers, where the CPGs were located, their aim, their objective/ purpose, who identified and interpreted the evidence (evidence funnel), how the CPGs were constructed (methods), and whether references underpinned recommendations. Table 2 reports the intended end-users, the ways that the evidence was presented, and the overall AGREE II score for each CPG. Only two CPGs scored over 50% for overall methodological quality (Maternal Health 67%; PC101 58%).

**Table 1:** Summary of included SA PHC CPGs.

	*Developer*	*Location found*	*Stated Aim*	*Evidence funnel*	*Info on CPG Methods*	*CPG Purpose / Objective*	*References for recommendations*
Acute asthma in children	Prof Ass	Peer-reviewed local journal	√	Specialist Experts	√	Standardise care	√
Acute asthma in adults	Prof Ass*	Peer-reviewed local journal	√	Specialist Experts	√	Standardise care	√
COPD	Prof Ass*	Peer-reviewed local journal	√	Specialist Experts	√	Standardise care	√
Hypertension	Prof Ass*	Peer-reviewed local journal	√	Specialist Experts	NA	Management	√
Type 2 diabetes	Prof Ass*	Peer-reviewed local journal	√	Specialist Experts	NA	Improve healthcare delivery	√
EDL	NDoH**	Government publication	√	Specialist Experts	NA	Standardise practice	NA
IMCI	NDoH**	Government publication	NA	Badged WHO^&^, UNICEF^&&^	NA	Step-wise guide to practice	√ (Presume WHO^&^ /UNICEF^&&^ material)
Malaria prevention	NDoH**	Government publication	√	Experts	NA	Step-wise guide to practice	√ Bibliography
Malaria treatment	NDoH**	Government publication	√	Experts	NA	Guide to risk assessment and practice	√ Bibliography
Maternal care	NDoH**	Government publication	√	Expert	NA	Management	√ Available on request
TB in children	NDoH**	Government publication	√	NA	NA	Raise awareness	NA
TB in adults	NDoH**	Government publication	√	NA	NA	Management	NA
PC 101 (symptom based guidance)	NDoH**	Government publication	√	NA	NA	Standardise practices	√ Website for more information
HIV in children	NDoH**	Government publication	√	NA	NA	Guidance	NA
HIV adults	NDoH**	Government publication	√	NA	NA	Management	NA
PMTCT (HIV)	NDoH**	Government publication	√	NA	NA	Guidance	√

*Professional Association

**NDOH–National Department of Health

^&^ WHO—World Health Organisation

^&&^ UNICEF—United Nations International Children's Emergency Fund (better known now as United Nations Children's Fund)

√ indicates this information was provided in the guidance document

NA indicates that this information was not available in the guidance document

**Table 2:** Intended end-users, evidence presentation and AGREE II overall scores.

	End users	Algorithm	Description of symptoms	Text-based recommend-ations	Products	Overall AGREE II score (%)
Acute asthma children	GP* (private or public)		√**	√	Evidence-based summary recommendations	42
Acute asthma adults	GP (private or public)	√	√	√	Evidence-based summary recommendations	42
COPD	GP (private or public)		√	√	Evidence-based summary recommendations	33
Hypertension	GP (private or public)	√	√	√	Evidence-based summary recommendations	50
Type 2 diabetes	GP (private or public)	√ checklist	√	√	Evidence-based summary recommendations	33
EDL	Primary care doctors and nurses with prescribing rights		√	√	Prescribing support information	50
IMCI	Frontline nurses / medics	√	√	√	Decision-support tool & recommendations	42
Malaria prevention	Frontline nurses / medics		√	√	Recommendations	42
Malaria treatment	All involved in management of malaria	√	√	√	Decision-support tool & recommendations	50
Maternal	Frontline nurses/ medics	√	√	√	Decision-support tool & recommendations	67
TB in children	Frontline nurses / medics	√	√	√	Decision-support tool & recommendations	33
TB in adults	Professional health care workers	√	√	√	Decision-support tool & recommendations	33
PC 101	Frontline nurses / medics	√	√	√	Decision-support tool & recommendations	58
HIV in children	Health practitioners		√	√	Recommendations	42
HIV adults	health practitioners		√	√	Recommendations	33
PMTCT (HIV)	Frontline nurses / medics	√	√	√	Decision-support tool & Recommendations	42

*GP–General medical Practitioner

**√ indicates the types of evidence presentations in the guidance documents

### Comparing SA CPGs with international quality practices

We first considered the content and quality of the five SA PHC CPGs developed by professional associations, and published in two peer-reviewed professional association journals (SA Medical Journal (SAMJ) (2016–2017 impact factor 1.71) and Journal of Endocrinology, Metabolism and Diabetes of SA (JEMDSA) (no current impact factor listed)). The CPGs dealt with conditions of acute asthma in children (SAMJ), acute asthma in adults (SAMJ), COPD (SAMJ), hypertension (SAMJ) and Type 2 diabetes (JEMDSA) (See Table 1). Compared with the internationally-agreed elements of quality CPG construction (Studies 1 and 2 [14, 26]), these CPGs variably reported on how they identified their evidence sources, how the quality, believability and relevance of this evidence had been determined, how the recommendations they proposed had been generated, or how the grades of evidence strength had been determined (when they were reported). All five SA PHC CPGs included the names of contributors to CPG writing. They also provided reference lists, however there was no indication of how and why these particular references had been sourced, and not all recommendations were linked with references [13, 14]. Guidance was presented as text, summary recommendations and flowcharts.

We then considered the 11 CPGs which were developed and published by the SA National Department of Health (NDoH), and compared these with international CPG practices. These CPGs provided either condition-specific guidance (e.g. TB, HIV, malaria) or overall guidance for multiple conditions (e.g. EDL, PC 101, IMCI). There was variable information provided on the type and location of the evidence underpinning these CPGs, and no NDoH CPG outlined an evidence searching or appraisal process. Only two CPGs provided the names of experts involved in the writing. Guidance was presented as text, pictures, flow charts and/or decision-making prompts.

### Model construction

**Fig 2:**
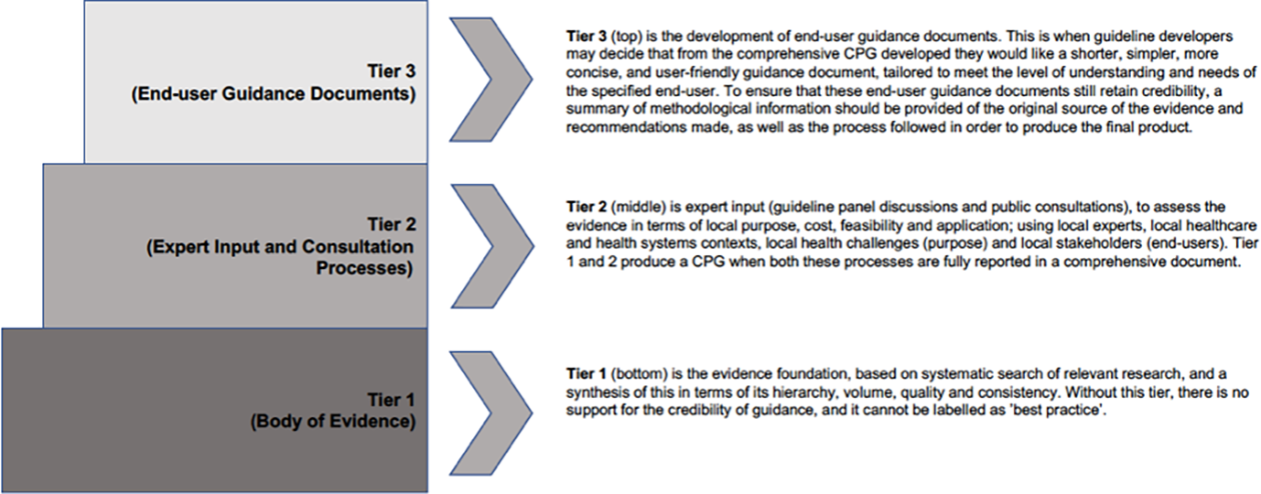
Conceptual model to improve efficient production of quality, locally-relevant CPGs in SA.

To address our purpose of improving efficient production of quality, locally-relevant CPGs in SA, we then developed a theoretical three-tiered model from the case study findings, to bridge the quality gaps between the SA PHC CPGs, and international CPG standards (Fig 2).

Tier 1 represents current, best-quality CPG recommendations, identified by systematic international searches for relevant CPGs that meet international quality construction standards [12–14]. Tier 1 provides best available information on ‘what to do’. We propose that without a robust Tier 1 underpinning it, no guidance document can legitimately be called a CPG.

Tier 2 describes evidence input from local experts, to put Tier 1 recommendations into context (‘how to do it locally’). This step potentially makes Tier 1 ‘what to do’ recommendations relevant to local evidence implementation, end-user needs, indigenous healthcare cultures, costs, feasibility, implementation barriers and patient engagement. Tier 2 draws on people with intimate knowledge of local health systems, contexts and challenges, as well as barriers and solutions to evidence implementation.

Tier 3 describes end-user guidance documents (evidence products to support ‘how to do it’). These operationalise the findings of Tiers 1 and 2, and are written specifically to assist end-users to implement evidence. Based on our case study findings, we propose a standard nomenclature for Tier 3 documents.

- *Evidence-based Summary Recommendations*. This term could be used where recommendations and explanatory text have been transparently extracted from good quality CPGs, layered with local contexts, and presented as resource material to support best-evidence diagnosis and management of specific conditions in specific environments. These documents may also provide prompts for healthcare providers such as pictures and text descriptions, summary recommendations for practice, checklists and/ or resource material. The SA professional associations’ CPGs are best classified as evidence-based summary recommendations (see as an example the SA Hypertension Society CPG [32]).
- *Patient Management Tools (PMTs)*. This describes patient management or decision-support tools designed for situations where frontline healthcare professionals have to make explicit, efficient and effective choices regarding diagnosis, immediate treatment and longer-term management. PMTs could variably include pictures and simple explanatory text, algorithms (‘if this, then do that’), symptom lists, management checklists, treatment choices and referral pathways. Many NDoH CPGs were presented as PMTs. An example of a PMT is PC 101 [33].
- *Protocols*. Protocols provide step-by-step guidance on ‘how to do’ specific healthcare tasks (taking bloods, resuscitation steps, diagnostic testing etc). Standard operating procedures may also be called protocols. Protocols may be embedded in Evidence-based Summary Recommendations and PMTs. We propose that a guidance document could be called a ‘protocol’ when there is only one correct way to complete a task, and this can be outlined by a step-by-step diagram or a series of text prompts.

## Discussion

To the best of our knowledge, this paper presents the first theoretical model designed to assist a resource-poor country to efficiently improve the quality of its CPGs.

The term ‘CPG’ is internationally recognised as referring to documents that meet international quality construction elements [14] and score well on all AGREE II quality appraisal domains [13]. On this basis, none of the included SA PHC CPGs should have been called CPGs, as they were, at best, guidance documents, supported by variable underpinning evidence. In many instances however, with little effort, the methodological quality (and hence believability) of these guidance documents could have been readily improved, by using the AGREE II principles as a construction framework. This would have prompted writers to describe how the underpinning evidence sources had been identified and critically appraised, and how recommendations were linked to references and strength of the body of evidence statements.

We propose that investing effort in producing country- or organisation-specific *de novo* CPGs (Tier 1) should be questioned in resource-constrained environments. The requisite rigorous evidence-searching required to establish Tier 1 is usually expensive and time-consuming [4, 9, 21]. ‘Recreating the evidence wheel’ (developing a new Tier 1) is inefficient, when information on ‘what to do’ is already available for many conditions, in current high quality CPGs written by experienced CPG developers elsewhere [4–8, 12–14]. Embracing the notion of ‘adopting’ existing CPGs produced by well-resourced developers (Tier 1) would decrease the impetus for resource-constrained countries or organisations to independently produce their own CPGs which outline ‘what to do’. It particularly seems reasonable to propose that for conditions where aetiology, symptoms and management are generally universal (such as asthma, hypertension, COPD, diabetes, cancer, HIV, TB, maternal health conditions, and many infant diseases), ‘what to do’ evidence from existing, current high quality CPGs could be readily adopted as Tier 1 information in resource-constrained environments. The source (adopted) CPGs should be referenced and cited appropriately [12–14], and the methods by which the source CPGs had been identified, assessed and applied should be available to end-users if required (e.g. on a freely-available website).

We further contend that the acknowledged CPG focus in resource-constrained environments should be on local implementation, and not evidence-construction. Thus by carefully layering already available Tier 1 recommendations with expert input and local contexts, barriers to evidence implementation could be efficiently identified and addressed (Tiers 2 and 3) [15–19, 21, 34]. Our case study identified that important information is required to ensure the credibility and transparency of the Tier 2 process. This includes contributors’ names and qualifications, how contributors were identified, their roles in producing the guidance document, their professional affiliations, their conflicts of interest and how these were managed, and how the Tier 2 discussions were managed [14, 30].

Based on our case study findings, Tier 3 documents should be viewed as evidence products, as they are designed for specific purposes and end-users, aim to improve and standardise local healthcare practices, and be auditable. Many SA PHC CPGs provided innovative Tier 3 documents which succinctly presented evidence summaries for specific end-user needs, thus providing step-by-step information on ‘how to locally apply’ Tier 1 ‘what to do’ recommendations. They provided a range of ways of fitting evidence to local contexts, cultures, practices and settings, and they presented recommendations in ways that were simple, easy-to-read and navigate. We believed that the SA PHC CPG Tier 3 products reflected the highest scoring AGREE II domains of clarity of presentation, and scope and purpose [13, 30], as they appeared to have been developed with specific purposes, end-users, needs and care settings in mind. These Tier 3 products would potentially be of interest to other resourced-constrained environments with similar PHC implementation challenges. However, without underpinning Tiers 1 and 2 (Fig 2), Tier 3 documents have little credibility. To ensure credibility of Tier 3 documents, Tier 1 information should be appropriately referenced, and a summary should be available of the Tier 2 processes.

**Fig 3:**
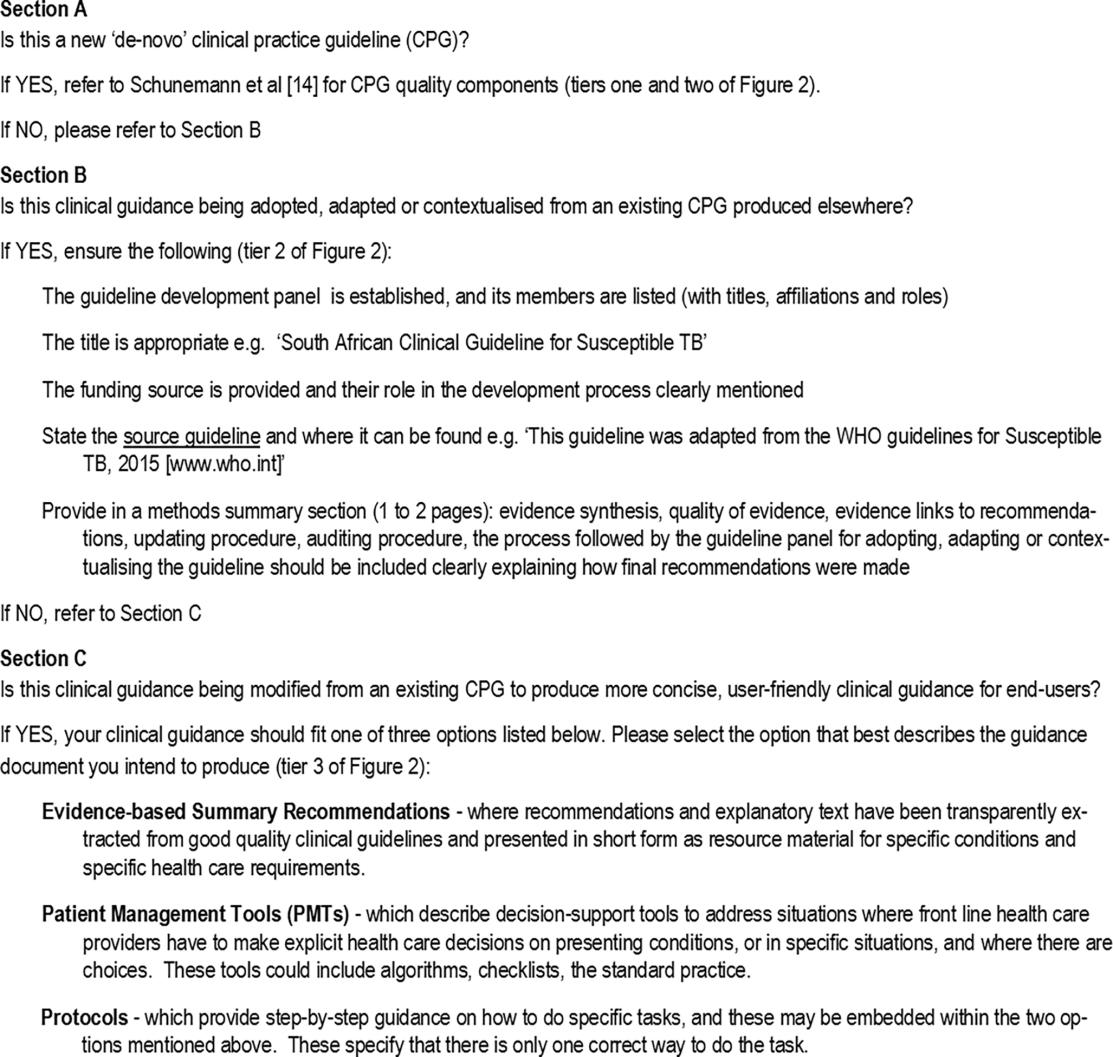
Checklist for classification of guidance documents.

In order to facilitate standard classification of, and terminology in, clinical guidance documents, and to maintain standard reporting practices that meet international requirements for CPG credibility, we developed a checklist to assist CPG writers in resource-constrained environments to identify in which category their guidance document belongs, as well as the requirements for each category (Fig 3). We also produced a glossary of common terms and explanations to assist CPG writers in resource-constrained environments (See S3 Appendix). Our model and the checklist require further testing to confirm its validity, and to test its relevance and utility in other environments which are not sufficiently resourced to efficiently independently develop Tier 1 evidence.

## Conclusion

This research demonstrated that the quality of SA PHC CPGs could be readily improved by using available high quality CPGs, referencing them appropriately, and focusing scant resources on local implementation. We believe that our three-tiered theoretical model (including proposed Tier 3 nomenclature), checklist and glossary of terms presents a novel resource to underpin efficient high-quality CPG writing activities in resource-constrained environments, and where there is an urgent need for best-evidence implementation to improve local healthcare processes and outcomes. We have confidence that these resources have relevance worldwide, particularly in situations where limited resources could be better spent on putting available evidence into effective practice, and not on ‘recreating’ an already existing evidence-base.

## Supporting information

S1 Appendix Good Reporting of A Mixed Methods Study (GRAMMS) reporting framework. (DOCX)

S2 Appendix MEDLINE search strategy (Ovid MEDLINE 1990 –Sept 2014). (DOCX)

S3 Appendix Glossary of terms. (DOCX)
