# Supplementary material for: Next generation clinical guidance for primary care in South Africa – credible, consistent and pragmatic
Source: PLoS One. 2018 Mar 30;13(3):e0195025. doi: 10.1371/journal.pone.0195025 (PMC5877861; doi:10.1371/journal.pone.0195025)
Supplement: S1 Appendix — (DOCX) [file pone.0195025.s001.docx]

S1 Appendix. Good Reporting of A Mixed Methods Study (GRAMMS) Reporting Framework [23, 25]

| **Items** | **Where addressed** |
| --- | --- |
| data collection methods | Lines 112, 122 |
| sequencing | Described as Studies 1-4 in text and in Figure 1 |
| sampling | Lines 114, 121-146, 137, 146-149 |
| priority of data | All equally weighted |
| points of integration | Figure 1 |
| data analysis techniques | Lines 153-159 |
