# Supplementary material for: Next generation clinical guidance for primary care in South Africa – credible, consistent and pragmatic
Source: PLoS One. 2018 Mar 30;13(3):e0195025. doi: 10.1371/journal.pone.0195025 (PMC5877861; doi:10.1371/journal.pone.0195025)
Supplement: S2 Appendix — (DOCX) [file pone.0195025.s002.docx]

S2 Appendix. MEDLINE Search Strategy (Ovid MEDLINE 1990 – Sept 2014)

| Concepts | Strategy | Hits |
| --- | --- | --- |
| 1 | ‘clinical practice guideline’ OR ‘practice guideline’ OR ‘guideline’ OR ‘clinical guideline’ OR ‘clinical guidance’ OR ‘guidance’ OR ‘practice guidance’ OR ‘clinical protocol’ OR ‘clinical decision-making’ OR ‘clinical recommendations’ OR guide& | 25263 |
| AND 2 | ‘develop&’ OR ‘writ&’ OR ‘construct&’ or ‘*de novo*’ OR ‘new’ OR evidence sum& OR manual OR ‘how to’ | 17117 |
| AND 3 | ‘qual&’ OR method& OR ‘best pract&’ OR ‘princip&’ or agreed OR struct& OR instruct& OR process& OR professional staff committee& OR consensus | 7211 |
