# Supplementary material for: Next generation clinical guidance for primary care in South Africa – credible, consistent and pragmatic
Source: PLoS One. 2018 Mar 30;13(3):e0195025. doi: 10.1371/journal.pone.0195025 (PMC5877861; doi:10.1371/journal.pone.0195025)
Supplement: S3 Appendix — (DOCX) [file pone.0195025.s003.docx]

S3 Appendix. Glossary of Terms

| Objective of Clinical practice guideline (CPG) | Why the CPG was developed and what its intended outcomes are. |
| --- | --- |
| Standard of practice | Practical benchmarks to guide and measure how health care is provided. |
| Step-wise guide to practice | Decision-making guides to assist in making correct healthcare decisions. |
| Guidance | Suggested management of a disease or condition. |
| Standard of care | Agreed level at which health services should be delivered. These should be underpinned by principles that aim to improve the quality of health service processes and subsequently improve health outcomes. |
| Decision-making tool | A document or instrument that includes all necessary options to assist in the process of making a decision. |
| Recommendations | Statements intended to optimise patient care and assist health care practitioners to make decisions about appropriate health care for specific circumstances. |
| Support information | Background information or underpinning evidence that assists healthcare providers to make decisions. |
| Scope and Purpose | Overall aim of the CPG, the specific health questions, and the target population [13]. |
| Stakeholder involvement | The extent to which the CPG was developed by the appropriate stakeholders and represents the views of its intended users [13]. |
| Rigour of development | The process used to gather and synthesize the evidence, the methods to formulate the recommendations, and to update them [13] |
| Clarity of presentation | The language, structure, and format of the CPG [13] |
| Applicability of the CPG to its intended setting | The likely barriers and facilitators to implementation, strategies to improve uptake, and resource implications of applying the CPG [13] |
| Editorial independence | The formulation of recommendations not being unduly biased with competing interests [13]. |
| Checklists | A list of items for checking whether tasks have been completed. |
| Algorithms | Step-by-step decision support graphics. May take binary form to support a judgement e.g. If Yes, then do this, If No, then do that documents. |
| Text-based recommendations | Recommendations and explanatory text have been transparently extracted from good quality CPGs with relevant contextual information often provided, and presented in short form as resource material for specific conditions and specific health care requirements. |
| Protocols | Documents that provide step-by-step guidance on how to do specific tasks (do this, then do that). |
| Patient management tools (PMTs) | Decision-support tools designed to address situations where frontline healthcare professionals have to make explicit healthcare decisions on presenting conditions, or in specific situations, and where there are choices. These tools could include algorithms, referral pathways, checklists, or recommendations for practice, with as much explanatory or contextual information as required regarding the various options the healthcare professional might decide to take for that particular patient. |
